# The Global Cohort of Doctoral Students: Building Shared Global Health Research Capacity in High-Income and Low- and Middle-Income Countries

**DOI:** 10.5334/aogh.3160

**Published:** 2021-01-25

**Authors:** Semira Abdelmenan, Christopher T. Andersen, Fentabil Getnet, Hari S. Iyer, Kesaobaka Molebatsi, Simone Passarelli, Sara M. Sauer, Muhammed Semakula

**Affiliations:** 1 Institute of Public Health, College of Medicine and Health Sciences, University of Gondar, Gondar, Ethiopia; 2 Addis Continental Institute of Public Health, Addis Ababa, Ethiopia; 3 Department of Epidemiology, Harvard T. H. Chan School of Public Health, Boston, United States of America; 4 School of Public Health, Haramaya University, Harar, Ethiopia; 5 Division of Population Sciences, Dana-Farber Cancer Institute, Boston, United States of America; 6 Department of Statistics, University of Botswana, Gaborone, Botswana; 7 Botswana Harvard AIDS Institute Partnership, Gaborone, Botswana; 8 Department of Nutrition, Harvard T. H. Chan School of Public Health, Boston, United States of America; 9 Department of Biostatistics, Harvard T. H. Chan School of Public Health, Boston, United States of America; 10 African Center of Excellence in Data Science, University of Rwanda, Kigali, Rwanda; 11 Centre for Statistics, Hasselt University, Hasselt, Belgium

## Abstract

Doctoral students in high- and low-income countries pursuing careers in global health face gaps in their training that could be readily filled through structured peer-learning activities with students based at partnering institutions in complimentary settings. We share lessons learned from the Global Cohort of Doctoral Students, a community of doctoral students based at the Harvard T. H. Chan School of Public Health, Haramaya University, University of Gondar, University of Botswana, and University of Rwanda College of Medicine and Health Sciences. Students in the Global Cohort program engage in collaborative research, forums for constructive feedback, and professional development activities. We describe the motivation for the program, core activities, and early successes.

## INTRODUCTION

Global health professionals require a wide range of management, analytic, and communication skills to conduct high quality research [1,2,3]. Yet few global health graduate degree programs offer classes in all of these areas, resulting in gaps in skills for students in high-income countries (HICs) and low- and middle-income countries (LMICs). Half of the respondents of a United States-based survey of recent global health graduates reported training gaps, particularly in project design and implementation, team building, and communication [4]. In LMICs, students often lack formal opportunities to build statistical computing and writing skills [5,6]. Bringing together students in HICs and LMICs with complementary skills provides an opportunity to build shared research capacity [7]. Doctoral students in high- and low-income countries pursuing careers in global health face gaps in their training that could be readily filled through structured peer-learning activities with students based at partnering institutions in complementary settings. We share lessons learned from the Global Cohort of Doctoral Students, a community of doctoral students based at the Harvard T. H. Chan School of Public Health, Haramaya University, University of Gondar, University of Botswana, and University of Rwanda College of Medicine and Health Sciences.

Though others have acknowledged the value of bidirectional global health exchanges [8,9,10], established global health research capacity building programs have generally not emphasized bidirectional learning for students [11,12,13]. One existing research capacity building approach allocates funds from HICs, allowing LMIC investigators to pursue degree programs or attend short courses at either HIC or LMIC institutions. While these training programs advance technical skills for individual beneficiaries in LMICs, concerns include financial sustainability, brain drain, and inadequate holistic professional development [14,15,16]. Another approach involves HIC investments in Centers of Excellence at LMIC institutions with potential for high research productivity, including North-South partnerships [13,17]. Many have critiqued these efforts for being driven by the research priorities of HIC rather than LMIC institutions [12,13,18]. Regardless of approach, nearly all capacity building programs emphasize development of skills among LMIC investigators, but do not explicitly aim for HICs investigators to learn from their LMIC colleagues [5,12,13,19]. The student-led Global Cohort of Doctoral Students (GCDS) was established in 2016 to address the gaps identified above by promoting reciprocal – rather than unidirectional – learning between doctoral students with global health research interests based at the Harvard T.H. Chan School of Public Health and partner institutions in LMICs.

### DESCRIPTION OF THE GLOBAL COHORT OF DOCTORAL STUDENTS PROGRAM

**Figure 1:**
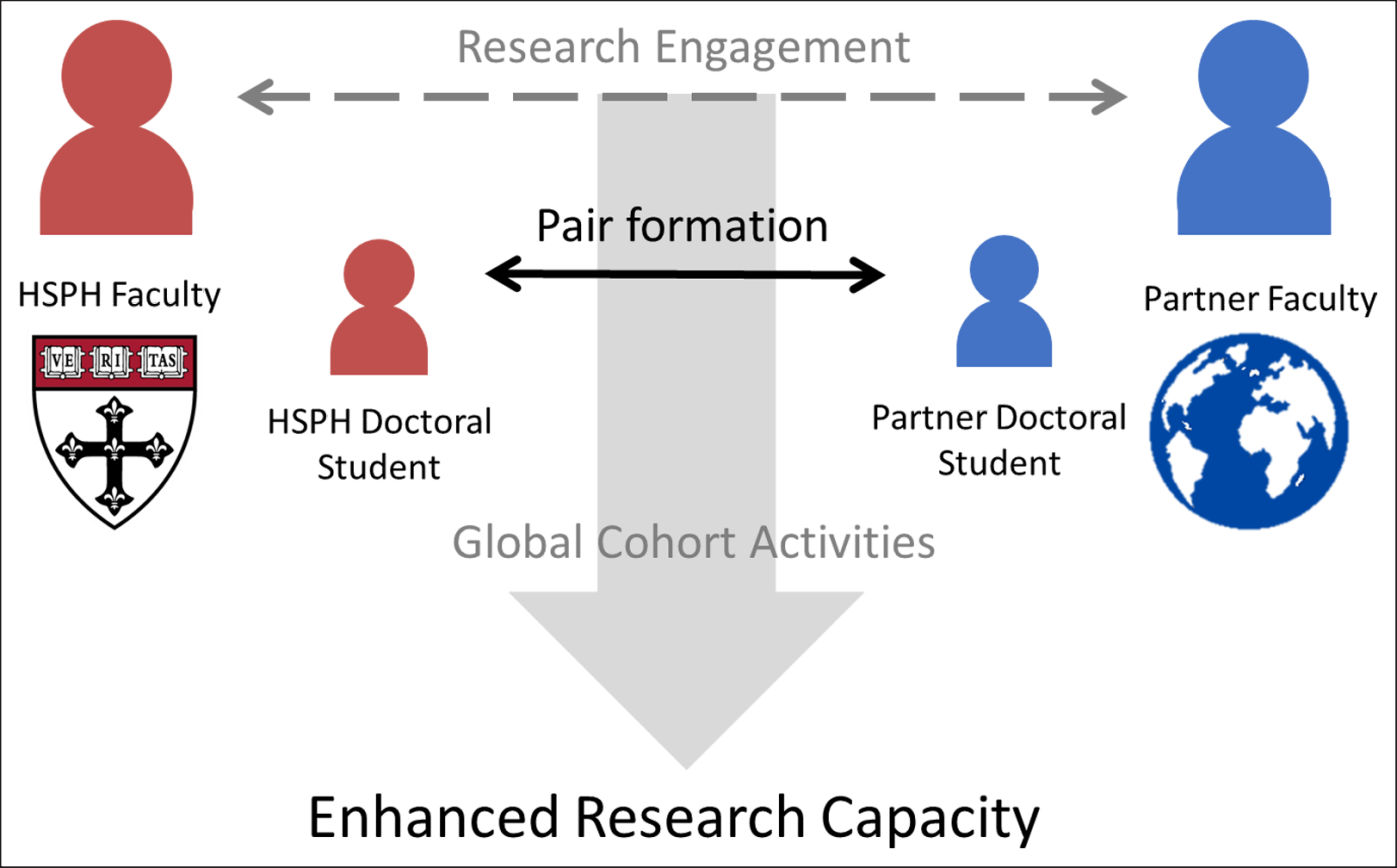
Foundation for Global Cohort Pair Formation. Abbreviations: HSPH, Harvard T. H. Chan School of Public Health.

GCDS is a community that promotes reciprocal learning between doctoral students around the world, promoting shared capacity to address public health problems by engaging in research, constructive forums for feedback, and professional development activities. Student pairs are formed based on shared research interests and projects between a doctoral student at the Harvard T. H. Chan School of Public Health and a student at a partner LMICs institution. The pairs receive mentoring by faculty with existing partnerships at the host institutions (***Figure 1***). GCDS students engage in three core activities: a learning contract, a web forum, and an annual conference. The learning contract is a mutual agreement, formed when pairs identify their common areas of interest and individual learning goals with input from faculty advisors from both institutions. To facilitate progress on their contract, each pair spends at least three weeks in the same location working on their deliverables. The second pillar of GCDS is the web forum, an online webinar that enables each member to share their work with other cohort members. On a rotating basis, one member shares a research presentation and solicits feedback from the rest of the group. This allows cohort members to receive constructive feedback as well as the opportunity for scientific learning across student pairs. The final core activity is an annual conference where all members of the Global Cohort gather in the same place to share knowledge, network, develop professional skills, and strategize about activities for the coming year.

## LESSONS LEARNED FROM THE THREE YEARS OF IMPLEMENTATION

We conducted an informal survey in which each pair of doctoral students was asked to provide details about (1) the bidirectional learning that occurred within their pair (and through their engagement with other pairs), (2) provide any examples of key successes achieved by pairs (including research outputs, learning goals, or other career development), and (3) provide key challenges encountered by the pair while completing their work. This survey enabled us to gather specific details regarding aspects of the program that succeeded and were less successful in promoting shared research capacity. Details about feedback are provided below with specific quotes provided in ***Box 1***.

**Box 1 Quotes describing GCDS member experiences regarding bidirectional learning, successes, and challenges.**

**BIDIRECTIONAL LEARNING**

- In data-rich environments, it is easy to fall into the trap where methods and data drive hypotheses. Learning about my partner’s research allowed me to see how important it was for public health problems to drive methods development, and how impactful those findings could be.” – *Harvard student, USA*

**SUCCESSES**

- At the time I joined the GCDS program; my partner from Harvard was skillful in statistical analysis and modeling while I had experience in designing studies to collect primary data. Teaming up with my partner helps to me to excel in statistical analysis, and to share my experience to him. Bringing our skills together helped us to publish 1 paper, complete 1 manuscript and develop a proposal to collect primary data” – *Haramaya Student, Ethiopia*

**CHALLENGES**

- As PhD students, time management became the key challenge to completing our agreed upon pair work. Therefore, it is crucial to have PhD supervisors on board to support the bidirectional research activities being a part of PhD duties.” – *University of Rwanda student, Harvard student*

### BIDIRECTIONAL LEARNING

Members of the GCDS program reported numerous examples of bidirectional learning within and between cohort pairs. Opportunities for shared learning within pairs occurred at exchange visits at each other’s institutions. These visits allowed students to build personal and professional relationships, engage in cross-cultural exchange, and receive and provide feedback on one another’s PhD projects. When Harvard students traveled to African partner sites, this learning occurred within the pair, but when partnering African students traveled to Harvard for the annual conference, learning occurred between pairs.

GCDS participants also reported that virtual seminars held every six weeks and the conference held in Boston, MA, USA provided opportunities for bidirectional learning across pairs. Virtual seminars provided each student the opportunity to present their work and provide and receive feedback from peers. Students found it helpful to present to scientists outside of their immediate research area because the feedback helped to improve their ability to communicate their work to a broader public health audience. During the conference in Boston, each GCDS member facilitated a training session on a selected topic based on his/her knowledge and experience. The training sessions covered technical skills (survey methods, qualitative methods, infectious disease epidemiology, and spatial methods) and professional development skills (grant writing and public speaking). Spending time together over the course of the conference allowed students to learn about each other’s skills, expertise, and career goals. The conference also strengthened social and professional networks between students based in Botswana, Ethiopia, Rwanda, and the United States.

While many skills gained from participating in GCDS, particularly for professional development, were similar among all GCDS participants regardless of home institution, there were some notable differences. HIC members reported that participation in GCDS provided them with experience in designing and managing field data collection processes in LMICs, designing implementation studies focused on the needs of low-income and marginalized communities, and understanding the health systems of LMICs. Members from LMICs reported that GCDS allowed them to develop advanced skills in data analysis including programing simulation in R, coding and interpretation of qualitative data, mastering statistical packages like Stata, and receiving feedback from peers on applications for postgraduate fellowships.

### SUCCESSES

The GCDS program has enabled PhD students from HICs and LMICs to realize early career scientific contributions through collaborative efforts. So far, two pairs (one in Botswana and one in Ethiopia) have published research articles in peer-reviewed journals [20,21]; three pairs have developed research manuscripts; one pair (Ethiopia) developed a proposal to collect primary data on delay in presentation of TB cases and subsequent effects on household transmission that was later awarded by the World Health Organization; and one pair, with support from Harvard University and the University of Rwanda, conducted two courses on Quantitative Methods for Monitoring and Evaluation and simulations in R for lecturers, PhD students, and Masters students and employees at the University of Rwanda.

### CHALLENGES

Despite acknowledgement of the opportunities for bidirectional learning and several successes, all groups reported challenges. The key challenges faced in the partnerships were adhering to timelines to complete work within the pairs and a lack of funding for African students to support travel and living costs to enable pair to work in-person. The challenge faced by all GCDS pairs was balancing the time spent on GCDS activities with the formal requirements of each student’s doctoral program. None of the students had a bi-directional exchange explicitly included in their doctoral training program, and so any time committed to GCDS activities had to occur outside of the formal dissertation and training. Consequently, it became difficult for them to prioritize GCDS projects. Another major challenge facing the long-term sustainability of the program is the need to clearly define the roles and expectations of the students once they graduate from their doctoral programs because the pairs continue to work on their shared research projects after graduation. Given the many benefits reported by GCDS that come from participating in a bidirectional training program, institutions should consider providing incentives through formal doctoral curricula to enhance the success of the program.

## CONCLUSION

GCDS has grown steadily through existing research partnerships between Harvard Chan and academic institutions in LMICs. Implementation of seven learning contracts is currently underway, spanning the United States, Botswana, Brazil, China, Ethiopia, and Rwanda. Pairs have engaged in collaborative training programs in statistics and data science in Ethiopia, Rwanda, and Botswana and have hosted a seminar on research capacity building in global health in Boston. Research accomplishments include obtaining a competitive research grant and two published manuscripts [20,21]. Finally, the Global Cohort’s work was recognized with a student prize at the Consortium of Universities during Global Health’s 10^th^ meeting. These achievements demonstrate that public health schools in the Global North and South see value in providing their students with opportunities to engage in bidirectional programs.

Graduates from global health training programs in HICs and LMICs often leave without acquiring important skills needed to conduct more effective and impactful global health research. GCDS addresses these gaps by promoting shared global health research capacity building between HIC and LMIC students. We hope that all universities offering global health training programs will consider offering similar opportunities to promote reciprocal learning among their students.

## References

[1] Sawleshwarkar S, Negin J. A review of global health competencies for postgraduate public health education. Front Public Health. 2017; 5(46). DOI: 10.3389/fpubh.2017.00046. eCollection. PMC5357810 28373970

[2] Ablah E, Biberman DA, Weist EM, et al. Improving global health education: Development of a global health competency model. Am. J. Trop. Med. Hyg. 2014; 90(3): 560–5. DOI: 10.4269/ajtmh.13-0537 24445206 PMC3945704

[3] Calhoun JG, Spencer HC, Buekens P. Competencies for global health graduate education. Infect Dis Clin North Am. 2011; 25(3): 575–92. DOI: 10.1016/j.idc.2011.02.015 21896359

[4] USAID. The Global Health Recent Graduate Study. https://www.cugh.org/sites/default/files/Global%20Health%20Recent%20Graduate%20Study%20Report_Final%20GHFPII%20Edits2.7.18.pdf Accessed September 9, 2019.

[5] Chu KM, Jayaraman S, Kyamanywa P, Ntakiyiruta G. Building research capacity in africa: Equity and global health collaborations. PloS Medicine. 2014; 11(3): e1001612 DOI: 10.1371/journal.pmed.1001612 24618823 PMC3949667

[6] Langer A, Diaz-Olavarrieta C, Berdichevsky K, Villar J. Why is research from developing countries underrepresented in international health literature, and what can be done about it? Bull World Health Organ. 2004; 82(10): 802–3. 15643806 PMC2623037

[7] Schriver M, Cubaka VK, Kyamanywa P, et al. Twinning Ph.D. students from south and north: towards equity in collaborative research. Educ Prim Care. 2015; 26: 349–52. DOI: 10.1080/14739879.2015.1079970 26808804

[8] Crump JA, Sugarman J. Working group on ethics guidelines for global health training (WEIGHT). Am J Trop Med Hyg. 2010; 83(6): 1178–82. DOI: 10.4269/ajtmh.2010.10-0527 21118918 PMC2990028

[9] Kolars JC, Cahill K, Donkor P, Kaaya E, Lawson A, Serwadda D, Sewankambo NK. Perspective: partnering for medical education in Sub-Saharan Africa: seeking the evidence for effective collaborations. Acad Med. 2012; 87(2): 216–20. DOI: 10.1097/ACM.0b013e31823ede39 22189887

[10] Arora G, Russ C, Batra M, Butteris SM, Watts J, Pitt MB. Bidirectional exchange in global health: Moving toward true global health partnership. Am J Trop Med Hyg. 2017; 97(1): 6–9. DOI: 10.4269/ajtmh.16-0982 PMC5508910 28719333

[11] Brieger WR, Adeniyi JD, Parker KA, Oladepo O. Health education in Africa: 1975-2000. Health Education Research. 2000; 15(4): 383–91. DOI: 10.1093/her/15.4.383 11066456

[12] Beran D, Byass P, Gbakima A, Kahn K, Sankoh O, Tollman S, Witham M, Davies J. Research capacity building – obligations for global health partners. Lancet Glob Health. 2017; 5(6): e567–68. DOI: 10.1016/S2214-109X(17)30180-8 28495256

[13] Franzen SR, Chandler C, Lang T. Health research capacity development in low and middle income countries: reality or rhetoric? A systematic meta-narrative review of the qualitative literature. BMJ Open. 2017; 7(1): e012332. DOI: 10.1136/bmjopen-2016-012332 PMC5278257 28131997

[14] Atickem A, Stenseth NC, Fashing PJ, et al. Build science in africa. Nature. 2019; 570(7761): 297–300. DOI: 10.1038/d41586-019-01885-1 31217608

[15] Pang T, Lansang MA, Haines A. Brain drain and health professionals. BMJ. 2002; 324: 499 DOI: 10.1136/bmj.324.7336.499 11872536 PMC1122434

[16] Bodnar BE, Claassen CW, Solomon J, Mayanja-Kizza H, Rastegar A. The effect of a bidirectional exchange on faculty and institutional development in a global health collaboration. PloS One. 2015; 10(3): e0119798 DOI: 10.1371/journal.pone.0119798 25799567 PMC4370667

[17] Nwaka S, Ochem A, Besson D, et al. Analysis of pan-African Centres of excellence in health innovation highlights opportunities and challenges for local innovation and financing in the continent. BMC Int Health Hum Rights. 12(11). DOI: 10.1186/1472-698X-12-11 PMC3492037 22838941

[18] Hedt-Gauther B, Airnhihenbuwa CO, Bawah AA et al. Academic promotion policies and equity in global health collaborations. Lancet. 2018; 392(10158): 1607–1609. DOI: 10.1016/S0140-6736(18)32345-6 30496066

[19] Trostle J, Simon J. Building applied health research capacity in less-developed countries: problems encountered by the ADDR project. Soc. Sci. Med. 1992; 35(11): 1379–87. DOI: 10.1016/0277-9536(92)90041-N 1462177

[20] Iyer HS, Kohler RE, Ramogola-Masire D, et al. Explaining disparities in oncology health systems delays and stage at diagnosis between men and women in Botswana: A cohort study. PloS One. 2019; 14(6): e0218094 DOI: 10.1371/journal.pone.0218094 31170274 PMC6553768

[21] Getnet F, Demissie M, Worku A, et al. Determinants of patient delay in diagnosis of pulmonary tuberculosis in somali pastoralist setting of Ethiopia: A matched case-control study. Int J Environ Res Public Health. 2019; 16(18): 3391 DOI: 10.3390/ijerph16183391 PMC6765848 31547479

